# Obesity as a Neuroendocrine Reprogramming

**DOI:** 10.3390/medicina57010066

**Published:** 2021-01-13

**Authors:** Abdelaziz Ghanemi, Mayumi Yoshioka, Jonny St-Amand

**Affiliations:** 1 Department of Molecular Medicine, Faculty of Medicine, Laval University, Québec, QC G1V 0A6, Canada; abdelaziz.ghanemi@crchudequebec.ulaval.ca; 2 Functional Genomics Laboratory, Endocrinology and Nephrology Axis, CHU de Québec-Université Laval Research Center, 2705 Boul. Laurier, Québec, QC G1V 4G2, Canada; mayumi.yoshioka@crchudequebec.ulaval.ca

**Keywords:** obesity, neurology, endocrinology, neuroendocrine, reprogramming

## Abstract

Obesity represents a health problem resulting from a broken balance between energy intake and energy expenditure leading to excess fat accumulation. Elucidating molecular and cellular pathways beyond the establishment of obesity remains the main challenge facing the progress in understanding obesity and developing its treatment. Within this context, this opinion presents obesity as a reprogrammer of selected neurological and endocrine patterns in order to adapt to the new metabolic imbalance represented by obesity status. Indeed, during obesity development, the energy balance is shifted towards increased energy storage, mainly but not only, in adipose tissues. These new metabolic patterns that obesity represents require changes at different cellular and metabolic levels under the control of the neuroendocrine systems through different regulatory signals. Therefore, there are neuroendocrine changes involving diverse mechanisms, such as neuroplasticity and hormonal sensitivity, and, thus, the modifications in the neuroendocrine systems in terms of metabolic functions fit with the changes accompanying the obesity-induced metabolic phenotype. Such endocrine reprogramming can explain why it is challenging to lose weight once obesity is established, because it would mean to go against new endogenous metabolic references resulting from a new “setting” of energy metabolism-related neuroendocrine regulation. Investigating the concepts surrounding the classification of obesity as a neuroendocrine reprogrammer could optimize our understanding of the underlying mechanisms and, importantly, reveal some of the mysteries surrounding the molecular pathogenesis of obesity, as well as focusing the pharmacological search for antiobesity therapies on both neurobiology synaptic plasticity and hormonal interaction sensitivity.

The nervous and the endocrine systems are the main regulators of the various homeostatic functions, including digestion, energy metabolism, cellular replication, tissues renewal, and fluids circulations. However, following selected factors (diet, intoxications, etc.) or internal changes (pathogens, cancer, etc.) related to both environmental impacts and genetic factors, these regulatory properties might lose their efficiency, or their balancing pathways may be reshaped. Within this context, energy homeostatic patterns are governed by diverse signals exchanged mainly between the control centers and the metabolic tissues (mainly adipose tissues, muscles, and the liver). This results in a balance between the energy intake and energy expenditure. However, under the influence of exogenous stimuli, such as an increased caloric intake, combined with a sedentary lifestyle (reduced energy expenditure) or certain therapeutic interventions, these regulatory mechanisms lose their ability to maintain the balance, and, therefore, the energy homeostasis is broken [1]. Such broken balance results in the development of obesity, with all its consequences on health at different tissular levels [2], following the accumulation of the excessive energy storages within specific tissues and locations. 

It is widely accepted that biology gave to mammals the ability to store energy as an advantage to make full usage of the available calories during the period of food abundance in order to survive the periods of food shortage and hunger. Hibernating animals (a status of deep physiological and metabolic changes [3,4,5,6]) are among the best illustrative examples of this property, as they store enough lipids within their adipose tissues prior to the hibernating period. Lipids are the nutritive elements with the highest caloric density, making them the best form of energy biostorage. This last property is confirmed by the fact that, unlike glucose, the lipids represent a weak stativity signal (“undetected” by centers sending signals to stop food intake), which increases their ratio within the food amount. Lipids are extremely important for biological functions, such as thermoregulation, energy productions, cellular membrane structures, and caloric storages. Therefore, the negative impacts of lipids seems to start only with excessive fat accumulations or abnormal locations, such as ectopic deposits [7], leading to the known obesity consequences, including cardiovascular diseases, inflammation, and metabolic syndrome [2].

Obesity, as a health problem, is an extreme form of fat storage. This fat storage was initially an ability reflecting a biological adaptation to food availability in the surrounding environment. Obesity pathogenesis and mechanisms are full of mysteries, and most studies on obesity focus on its basic definition as excessive abnormal energy storage resulting from having an energy intake superior to energy expenditure. However, this metabolic broken balance [1] could be the outcome of obesity rather than its underlying mechanism, and the starting point could result from numerous neurological and endocrine changes. Indeed, the implications, effects, and interactions of the nervous and endocrine systems with the diverse organs and tissues involved in both obesity development and energy balance indicate a possible classification of obesity as a neurological diseases combined with endocrine abnormalities. 

Starting with the nervous system, the existence of brain centers that both receive signals form the digestive system (where the nutritive elements are detected) and controlling food intake support such a neuroendocrine approach. These signals include ghrelin [8], glucagon-like peptide 1 [9], and peptide YY [10], which act on centers including the hypothalamic melanocortinergic system [11]. These signals have been associated with diseases and complications linked to obesity such as diabetes [12], and they represent promising therapies for neurological diseases due to properties such as neurotrophic and neuroprotective actions [13]. This again shows the “neurological” character of these energy-metabolism signals. The concept of food addiction [14,15] in the context of obesity [16], with the neurobiological mechanism similar to drug abuse with the dopaminergic rewarding system [17], further highlights the neurological reprogramming that results from a neuroplasticity [18] of the involved neurons through functional and structural adaptation [19]. This aims to meet the novel neuronal activities required to adapt to obesity status. In addition, the existence of pharmacological approaches targeting the nervous system to treat obesity, such as utilization of the glucagon-like peptide-1 receptor [9,20], go beyond the usage of the classical obesity-related pillars; exercise and diet [21] further indicate that the nervous system’s involvement in obesity development. Coming back to the endocrine system, both insulin resistance [22] and leptin resistance [23] illustrate best how obesity status reprograms hormonal functions during obesity via modifying the interaction quality of energy balance-control hormones with their target tissues. 

Biology provides organisms (mainly mammals) with the ability to store energy as a mechanism to face possible hunger periods or adapt to a lack of food resources. However, with an increase in food resources, this biostorage ability, initially vital, could transform into a leading cause of obesity development. This ability of organisms to store energy (fat) makes it difficult to lose weight afterwards. Indeed, once an increased body weight/fat stores level is reached, it becomes the “new reference” toward which the metabolism and energy homeostasis are shifted in order to maintain or return to the newly set up point of fat storage level, thereby hindering weigh loss attempts. Within the context of brain involvement in energy control, this central new “reference” of body/fat weight could be explained through a neuroplasticity related to the centers controlling food intake and energy expenditure. This neuroplasticity could also occur in the peripheral neurons and, therefore, impact the innervations or the peripheral metabolic tissues. This is another illustration of the reprogramming of the neurological control of energy balance and how it contributes to obesity. For instance, the liver [24] and adipose tissues [25] are innervated by neurons that modify their metabolic activities. These properties explain the observed decrease in the basal metabolic rate seen with weight loss [26]. This decrease in the basal metabolic rate occurs to compensate for the reduced food intake or, more generally, the energy balance in order to prevent or limit weight loss, thereby predisposing individuals to weight regain. This property is a consequence of the setup of the new body/fat level reference, as described above, and is representative of the survival ability provided by biology to store energy to endure food-shortage periods. Similar to neuroplasticity, the modifications of receptor sensitivity, such as that of insulin receptors, indicates endocrine reprogramming that makes receptors in need of the strongest stimulations. This may also represent the need to store more energy to adapt to the obesity status of requiring more energy storage and, therefore, more insulin (and also more leptin, for which a resistance also develops during obesity). These calorie- and hormonal-centric explanations (neuroendocrine) also extend to the regulation of metabolic diseases like obesity. Indeed, it fits, for instance, with the hypothesis linking a high-fat diet with trefoil factor 2 (TFF2) as a lipid-induced signal for which the corresponding gene (*Tff2*) is induced by the high-fat diet [27], whereas its knockout provides protection from obesity [28] by leading to an antiobesity metabolic phenotype [29].

This concept of neuroendocrine reprogramming has as a particular outcome: the establishment of a novel energy balance status for optimum energy storage rather than limitation of caloric intake. Indeed, before obesity develops, satiety signals are strong enough to limit food intake, whereas once obesity is established, the hunger becomes “chronic” (reduced/inefficient satiety signals), leading to an increased food intake and more energy storage with less energy expenditure (metabolic slowing). Therefore, this reprogramming is the process via which the organisms shape their metabolic phenotype as an adaptation to the new status that obesity represents in terms of the need for increased energy intake and storage capacity. These neuroendocrine changes could explain obesity outcomes such as regeneration impairment [30] as well as the beneficial effects of exercise [31,32,33] and the molecules induced by exercise (e.g., [34,35,36]), beyond which there is exercise-induced neuroendocrine reprograming that shapes the metabolic phenotype and explains the importance of regular exercise in obesity therapy.

Elucidating the concepts surrounding the classification of obesity as a neuroendocrine reprogrammer could optimize the understanding of its underlying mechanisms and, importantly, reveal some of the mystery surrounding the molecular pathogenesis of obesity, as well as focusing the pharmacological search for antiobesity therapies on neurobiology synaptic plasticity and hormonal interaction sensitivity. 

## Data Availability

Not applicable.
